# Housing conditions in European one-person households

**DOI:** 10.1371/journal.pone.0303295

**Published:** 2024-05-17

**Authors:** Marlena Piekut

**Affiliations:** Warsaw University of Technology, Warsaw, Poland; Budapest Metropolitan University of Applied Sciences, HUNGARY

## Abstract

This study addresses the satisfaction of housing-related needs in single-person households across European countries. The primary objective is to assess the housing satisfaction of single-person households in European countries, specifically within the Visegrad Group. The study seeks to identify trends in housing conditions, create a ranking of countries based on these conditions, and categorize countries with similar levels of unmet housing needs. The study employs statistical measures and methods to achieve its objectives. Time series are constructed for European countries, and linear trends are analyzed to identify statistically significant changes in selected housing aspects from 2005 to 2022. Various research tasks, including ranking countries and grouping them based on housing conditions, are accomplished using established methods like linear ranking and Ward’s cluster analysis. Key findings include significant variations in financial burdens related to housing costs, thermal comfort, environmental pollution, and safety issues across European countries. The study reveals both improvements and challenges in housing conditions from 2005 to 2022 in one-person households. For instance, financial stress due to housing costs decreased in some countries, while thermal comfort issues improved in several nations. The results also highlight the heterogeneity within the Visegrad Group. The study concludes that there is a need for targeted actions to address housing-related issues in single-person households. The findings underscore the importance of investments in building energy efficiency, initiatives for affordable housing construction, and environmental policies. The research emphasizes the impact of housing conditions on health, well-being, and overall community life, urging policymakers to consider these factors for holistic improvement in the housing sector.

## Introduction

Appropriate housing conditions provide numerous benefits for individuals and society as a whole. Housing should offer a comfortable and convenient living space [1], which impacts the quality of sleep, rest, and overall mental well-being. Housing should be maintained to proper hygiene standards. The absence of moisture, mold, and other harmful factors can reduce the risk of diseases and health problems [2]. Housing provides individuals with privacy and autonomy [3]. It is a place where one can feel independent and control their environment. Housing also serves as a safe shelter from weather conditions, security threats, and other risks, which are crucial for health and well-being [4]. Good housing provides space for building family and social relationships. Having appropriate housing conditions can contribute to personal development. It is a place where one can nurture their interests, passions, and skills, which can positively impact individual productivity [5]. Good housing can influence education and work. Access to a quiet and well-equipped space for studying or remote work is essential for success in these fields. Housing can also affect one’s self-esteem and social respect. People living in decent conditions often feel more confident and respected by others [6]. Appropriate housing conditions are crucial for the overall well-being of individuals and impact many aspects of their lives. They provide safety, health, comfort, privacy, and many other benefits that enhance quality of life. Therefore, it is important to strive for access to decent housing conditions for all.

The aim of this study is to assess the satisfaction of housing-related needs in single-person households in European countries, with a particular focus on the Visegrad Group countries. The following research objectives have been set:

- To present the state of inadequacy in providing proper housing conditions in European countries and identify development trends in this area from 2005 to 2022.
- To create a ranking of countries based on housing conditions in single-person households.
- To identify groups of countries with similar levels of unmet housing needs in single-person households.

The subject of interest is single-person households, as they represent a specific group [7, 8], distinguished from other households by their full financial independence and responsibility for managing their finances. Individuals living alone often make decisions about purchases, home furnishings, travel, and other aspects of life independently of others. Single-person households make up a significant portion of households in countries. According to Eurostat data in 2022, in the Czech Republic and Hungary, single-person households accounted for nearly one-third of all households, in Poland, approximately one-fourth, and in Slovakia, almost every tenth household was composed of a single person [9].

The research focuses on unmet housing needs in single-person households. Examining the satisfaction of needs is important because understanding which needs are essential for people’s well-being is crucial for developing social and public policies. Governments can strive to satisfy basic needs such as housing to improve citizens’ quality of life. Analyzing the level of satisfaction of basic needs, including housing, can help identify areas where social inequalities and poverty exist. Recognizing unmet needs can serve as an impetus for policymakers to develop appropriate assistance programs. Entrepreneurs often create new products and services to satisfy new or existing consumer needs. Examining needs and consumer trends can drive innovation and economic development.

## Literature review

### Theories of human needs

A need is a fundamental human inclination or desire for something that is essential for the preservation of life, well-being, development, and satisfaction. Human needs play a pivotal role in shaping the behaviors, choices, and actions of individuals [10]. The satisfaction of a need means that an individual is capable of fulfilling or meeting that need in a manner that brings them satisfaction, comfort, or relief. Meeting needs can lead to feelings of contentment, peace, or a sense of fulfillment.

From a theoretical standpoint, it is assumed that there are various types of human needs that influence consumer behaviors and economic decisions. These include basic needs such as the need for food, shelter, clothing, and healthcare. Addressing these needs is often considered a priority in economic analysis because they are essential for survival and well-being. Some economic theories suggest that individuals strive to maximize pleasure (hedonism) and minimize pain. For instance, the utility theory is based on the assumption that consumers seek to maximize utility, which is the level of satisfaction or pleasure derived from consumption. Hence, hedonistic needs can be identified [11].

There are also psychological needs, such as the need for prestige, social recognition, autonomy, or a sense of belonging. Consumers may make consumption decisions to satisfy these needs, even if they are not directly related to material well-being.

Furthermore, culture and society influence which needs are considered important and how they are satisfied. Different cultures may have distinct standards and values that shape consumer behaviors.

Hierarchical needs can also be identified. Abraham Maslow’s Hierarchy of Needs theory suggests that people have hierarchically organized needs, starting from physiological and safety needs and then encompassing social, esteem, and self-actualization needs [12]. This theory is one of the most well-known psychological theories. Maslow developed this theory to understand what motivates people and guides their behaviors. Maslow’s theory describes five main levels of needs that are hierarchically arranged [13]. Physiological needs form the foundation of the needs pyramid. This is the lowest level of the hierarchy, encompassing basic needs such as food, water, sleep, warmth, and other elemental needs necessary for survival. If these needs are not met, they become the primary motivator for action. Slightly higher up are safety needs, which involve people’s desire for security and stability. These needs include the need for a safe shelter, employment, financial stability, and protection from threats. People aim to eliminate risk and uncertainty in their lives. The next level is the need for belongingness and love, focusing on the need to establish social relationships, love, acceptance, and group affiliation. People desire close relationships with family, friends, and life partners. Above in Maslow’s hierarchy are the needs for esteem and respect. At this level of needs, people seek social recognition, respect from others, and achievements in society. This pertains to the need for appreciation of one’s worth, professional accomplishments, and personal achievements. At the highest level of the hierarchy are self-actualization needs. This level encompasses the need to develop one’s full potential, pursue passions, engage in creativity, and achieve personal goals. Individuals at this level seek self-fulfillment and the realization of their dreams and life objectives.

According to Maslow, needs at lower levels of the hierarchy must be satisfied before an individual is motivated to pursue higher-level needs. This means that people initially focus on satisfying physiological and safety needs, followed by social, esteem, and self-actualization needs. In addition to Abraham Maslow’s Hierarchy of Needs theory, there are many other theories and concepts related to human needs. Clayton Alderfer expanded upon Maslow’s Hierarchy of Needs by creating the ERG model, which simplifies human needs into three categories: existence (similar to Maslow’s physiological needs), relatedness (similar to social and love needs), and growth (similar to esteem and self-actualization needs). This model is a type of motivation theory that focuses on the content of these needs. This theory is known as the "ERG theory" (Existence, Relatedness, Growth) [14].

David McClelland [15] identified three main types of needs in his theory: the need for achievement, the need for power, and the need for affiliation. These needs influence the motivation and behavior of individuals in both work and everyday life.

On the other hand, the self-determination theory by Richard M. Deci and Edward L. Ryan posits that people have three basic psychological needs: autonomy (feeling in control of one’s life), competence (feeling effective), and relatedness (feeling close and connected to others) [16, 17].

Manfred Max-Neef [18] proposed a model that considers nine fundamental human needs, such as subsistence, protection, affection, understanding, participation, leisure, creation, identity, and freedom. This theory emphasizes that meeting these needs is crucial for the well-being and development of individuals and societies.

Carol Ryff created a theory of six dimensions of psychological well-being, encompassing needs such as autonomy, personal growth, mastery, purpose in life, positive relations with others, and environmental mastery [19].

These theories vary in their approach to categorizing and classifying human needs and in their applications across different fields, including psychology, sociology, management, and economics. Each of these theories provides valuable insights into human needs and motivations and helps to better understand how they influence behaviors and choices.

The satisfaction of needs is a crucial element of an individual’s well-being and can contribute to their psychological and emotional satisfaction. Many theorists and psychologists, including Abraham Maslow, assume that the hierarchy of needs, from the lowest level (physiological needs) to the highest (self-actualization needs), influences an individual’s motivation and behavior, with the satisfaction of lower-level needs being a necessary condition for satisfying higher-level needs. It is worth noting that different individuals may have different priorities and ways of satisfying their needs, making the interpretation and fulfillment of needs an individualized process.

### Housing needs in the theory of needs

Abraham Maslow’s Hierarchy of Needs theory is one of the key frameworks for understanding human needs, including housing needs [20, 21]. Housing needs can be interpreted within the context of this hierarchy as follows. In the context of physiological needs, the lowest level of Maslow’s hierarchy, the need for housing corresponds to the need for shelter and a secure place to live [22]. People require a roof over their heads to protect themselves from extreme weather conditions and other physical threats. At the level of safety needs, the second level, people strive for a sense of security and stability. Housing is a critical element in meeting these needs as it provides a sense of protection and privacy [23, 24]. A safe and stable place of residence can offer psychological comfort and peace. Concerning the needs for belongingness and love, housing can serve as the center of family life, a space where social bonds and family relationships are formed. It is a place where people share their lives with others and experience emotional support. On the level of esteem and respect needs, people seek recognition and respect from society. Having suitable housing can influence social perception and social status [25]. Individuals with access to appropriate housing may have increased self-confidence and gain greater respect in society. Self-actualization needs represent the highest level of the hierarchy, where people aspire to develop their potential and achieve their own goals and passions. Housing can provide a space where people can cultivate their interests, passions, and create an environment that supports their personal growth [26]. In summary, in Maslow’s theory, housing needs can be interpreted as part of the lower levels of the hierarchy, such as physiological needs and safety needs. Housing is a fundamental element that provides people with shelter, security, and comfort, which are essential to fulfilling these basic needs. However, it can also be argued that housing fits into higher levels of the hierarchy, such as belongingness, esteem, and self-actualization needs, through its impact on social relationships, social status, and personal development.

Other need theories can also be related to housing needs. For example, McClelland’s theory can be applied to meeting housing-related needs. Individuals with a strong need for achievement may set challenges regarding their housing conditions, striving to obtain housing that represents their success and prestige. They may be willing to invest in homes or apartments with unique designs or significant social status. For them, enhancing their living environment, implementing innovative solutions, and continuously improving their interior can lead to satisfaction with their achievements [27]. Individuals with a strong need for affiliation may value housing that provides the opportunity to build close relationships with neighbors and the local community. Housing in friendly and social neighborhoods may be attractive to them. They may also prefer housing that allows for social and integration-oriented gatherings, contributing to building relationships with fellow residents. Conversely, individuals with a strong need for power may take it upon themselves to control their living environment. They may be more interested in owning large, manageable properties or apartments that allow them full control over their surroundings. For them, exercising their power to influence their living environment, such as through resident organizations or initiatives to improve their neighborhood, may be important. It’s worth emphasizing that preferences regarding housing conditions vary among individuals and depend on many factors, such as culture, personal values, financial situation, and more. McClelland’s theory can help understand why some people choose specific types of housing or why they attach importance to certain aspects of their living environment.

### Country rankings in terms of housing conditions

Housing conditions in different countries are reflected in international rankings. The indices used to create these classifications encompass various aspects related to the accessibility, quality, and sustainability of housing. Examples include the Better Life Index published by the OECD, which considers housing-related issues such as dwellings without basic facilities, housing expenditure, and rooms per person. Visegrad Group countries are positioned at relatively distant ranks in this particular ranking. On average, there are 1.1 rooms per person in housing in Slovakia and Poland (34th and 35th positions out of 41 in the OECD country ranking), while the Czech Republic has 1.5 rooms per person (27th position), indicating that Poles and Slovaks may live in rather crowded conditions. Overcrowded housing can have a negative impact on physical and mental health, relationships with others, and the development of children. Moreover, dense housing is often a sign of inadequate water and sewage infrastructure. In terms of private access to an indoor flushing toilet, the percentage varies from 96.55% in Hungary (29th position out of 41) to 99.51% in the Czech Republic (17th position out of 41). Housing costs constitute a significant portion of the household budget and represent the largest single expenditure for many individuals and families when considering elements such as rent, gas, electricity, water, furniture, and repairs. Households from the Visegrad Group, on average, spend from 19.9% in Hungary (16th position out of 41) to 27.4% in Slovakia (41st position out of 41) of their gross adjusted disposable income on housing costs [28], once again highlighting the challenging situation for Slovaks.

Rankings that consider sustainable housing aspects take into account issues such as energy efficiency, renewable energy sources, and recycling practices. In the Green Living Index for 2022, the Visegrad Group countries hold the following positions: Slovakia (9th place out of 28 EU countries), Hungary (19th), Czech Republic (25th), and Poland (27th). The Green Living Index utilizes official EU data for a comparative analysis. Its objective is to evaluate how European countries are addressing various challenges associated with transitioning to a more sustainable lifestyle [29].

Another ranking illustrating housing conditions, specifically satisfaction with them, is published by the European Institute for Gender Equality. Calculations are derived from microdata sourced from the European Quality of Life Survey (EQLS), coordinated by Eurofound. This survey aims to accurately represent the entire population of residents aged 18 or older in the surveyed country. The latest data published by the mentioned Institute relates to the year 2016. The highest satisfaction with housing conditions was recorded in Hungary (7.8 points compared to 7.7 for EU-28), followed by Slovakia (7.7 points). Lower values than the EU-28 average (7.7 points) were noted for Poland (7.3 points) and the Czech Republic (7.4 points) [30].

These rankings serve as significant research tools, allowing for the comparison of various aspects of housing conditions on an international and local scale. Analyzing these rankings can provide a deeper understanding of differences between countries and identify areas that require further research.

## Data and research methods

### The concept of housing conditions and variables used in the analysis

Housing conditions can be considered from various perspectives, taking into account different aspects (physical, economic, social, environmental, ecological, legal-spatial).

The physical aspect includes the technical Eurostat of the building, the size and layout of rooms, heating and ventilation, toilet and bathroom availability, and hygiene conditions. The economic aspect involves assessing affordability and prices of housing in the market, including rent, fees, and maintenance costs, as well as housing subsidies. The social aspect, on the other hand, pertains to housing accessibility for different social groups, the quality of relationships among residents, participation in local community life, neighborhood safety, and the risk of crime. The environmental aspect encompasses geographical location, housing’s proximity to natural environments, access to green spaces and parks, energy efficiency, the use of eco-friendly technologies, and environmental impact reduction, including environmental pollution. Housing conditions also include an ecological aspect, which considers energy, water, and resource consumption in the context of housing, as well as recycling practices and waste management in the area. Lastly, the legal-spatial aspect involves building regulations, tenant/owner rights and responsibilities.

Examining housing conditions from these various perspectives allows for a more comprehensive understanding of the factors influencing housing quality and accessibility, as well as the actions that can be taken to improve housing conditions for communities.

The variables used in the analysis were derived from the EU-SILC survey. Ten variables characterizing housing conditions were selected (listed in the table). Each variable indicated the percentage of one-person households characterized by a specific feature. The selected variables covered different aspects of housing conditions. A detailed assignment of variables to aspects of housing conditions is presented in the Table 1.

**Table 1:** Aspects related to housing conditions in households.

Aspect	Variable
**Economic**	housing cost overburden rate;
	persons who cannot afford to replace worn-out furniture;
	households with heavy financial burden due to the housing costs;
**Phisical**	total population living in a dwelling with a leaking roof, damp walls, floors or foundation, or rot in window frames or floor;
	total population having neither a bath, nor a shower in their dwelling;
	total population not having indoor flushing toilet for the sole use of their household;
	inability to keep home adequately warm;
**Social**	crime, violence or vandalism in the area;
	noise from neighbors or from the street
**Environmental**	pollution, grime or other environmental problems.

Source: own work

It should be noted that some variables may be related to several different aspects, e.g. the inability to keep the home adequately warm could also be assigned to the economic aspect.

### Data sources and study design

The study was focused on one-person households in European countries, with particular emphasis on Polish households. The data came from the Eurostat database. The section containing information on Income and living conditions was utilized, selecting the following variables for single-person households:

A-1. Housing cost overburden rate [online data code: TESSI166], B-2. Persons who cannot afford to replace worn-out furniture [ILC_MDHO07__custom_7431273], C-3. Population living in a dwelling with a leaking roof, damp walls, floors or foundation, or rot in window frames or floor [ILC_MDHO01__custom_7431302], D-4. Population having neither a bath, nor a shower in their dwelling [ILC_MDHO02__custom_7432308], E-5. Noise from neighbours or from the street [ILC_MDDW01__custom_7432326], F-6. Pollution, grime or other environmental problems [ILC_MDDW02__custom_7432344], G-7. Crime, violence or vandalism in the area [ILC_MDDW03__custom_7432374], H-8. Population not having indoor flushing toilet for the sole use of their household [ILC_MDHO03__custom_7432398], I-9. Households with heavy financial burden due to the housing costs [ILC_MDED05__custom_7432462], J-10. Inability to keep home adequately warm [ILC_MDES01__custom_7434289]. In the subsequent sections of the paper, alphanumeric notations for variables will be used in the tables, where, for example, A-1 represents the housing cost overburden rate.

To achieve the research objective and carry out the tasks presented in this paper, the researcher had to apply a variety of statistical measures and methods.

Research task no. 1. Identification of differences and changes in the satisfaction of needs related to selected housing conditions in European countries. Time series were constructed for European countries, which were then examined using linear trends to determine if there were statistically significant changes in the satisfaction of selected groups of needs between 2005 and 2020 or 2022. The analysis period (end year) depended on data availability. Due to the length constraints of the article, four variables describing various aspects of housing conditions were selected for detailed analysis in this section: households with a heavy financial burden due to housing costs (economic aspect), inability to adequately heat the home (physical aspect), crime, violence, or vandalism in the area (social aspect), and pollution, dirt, or other environmental problems (environmental aspect).

Using the α level (denoted as α or alpha), the probability of rejecting the true hypothesis is determined at the desired level. An alpha level of 0.05 was adopted in the paper.

Research Task No. 2. Ranking countries based on housing conditions in one-person households. To achieve this, the linear ranking method was used.

The linear ranking method was used to establish the order of countries (based on housing conditions in one-person households). The idea behind the linear ranking of multi-dimensional objects is based on the concept of a ranking binary relationship. From the axioms of this relationship, it is possible to determine which of two arbitrary objects in the set is the first (better) and which is the second (worse), as well as whether they are identical. In the process of linear ranking, the following steps are distinguished: determining the nature of the variables (stimulants, nominants, destimulants), assigning variable weights, variable normalization, determining pattern coordinates in the case of pattern aggregation, unconstrained or constrained aggregation [31]. In this study, all variables had a destimulant character, and the variables were normalized. The unconstrained aggregation method was chosen. Unconstrained linear ranking is useful in situations where clear comparative criteria or patterns are lacking. The ranking includes 10 variables for the most recent available year, either 2020 or 2022.

Research Task No. 3. Grouping countries based on the percentages of one-person households reporting unmet housing-related needs.

Cluster analysis using Ward’s method was employed for cluster delineation. The goal of Ward’s cluster analysis method is to group similar observations or cases into clusters in a way that minimizes the variance within clusters. This method is often used in cluster analysis of numerical data [32].

The following steps can be identified in Ward’s cluster analysis:

1. Cluster initialization: initially, each data point (or observation) is treated as a separate cluster.
2. Distance calculation: for each pair of clusters, the distance measure between points is calculated. In this study, the square of the Euclidean distance was used.
3. Cluster merging: a pair of clusters is selected for merging, which results in the smallest increase in the variance within clusters. The variance within a cluster measures the dispersion of data points within the cluster.
4. Cluster update: the merged clusters become a new cluster, and the entire process is repeated until only one cluster containing all observations remains.
5. Hierarchical structure creation: as clusters are merged, a hierarchical structure of clusters, known as a dendrogram, is created, depicting the sequence of cluster mergers.

The main feature of Ward’s cluster analysis method is its goal to minimize the variance within clusters, which means that clusters are as internally homogeneous and distinct from each other as possible. It is one of the agglomerative methods, which means that it starts with individual data points and gradually merges them into larger clusters. This method is particularly useful when analyzing data with certain assumptions about data distribution and when a hierarchical structure of clusters is desired [33].

In the case of cluster analysis, we have two essential assumptions: sample representativeness and collinearity. Lack of representativeness can lead to a distortion of cluster structures. The sample must be randomly selected to generalize the obtained results to the entire population. The data were sourced from the Eurostat database, and based on this, they can be considered representative.

Collinearity occurs when independent variables are strongly correlated. Its presence makes it challenging to assess the true impact of individual variables. In cluster analysis, collinearity can create an artificial cluster arrangement because collinear variables may have a greater influence on similarity measures (distances). The resulting cluster arrangement can be misleading. All data were expressed in percentages. The Pearson correlation coefficient was chosen as the measure. Due to collinearity, variable H-8 was not included in the final cluster analysis. Population not having a flushing toilet for the sole use of their household (r > 0.09) (Table 2).

**Table 2:** Correlation coefficient values for the analyzed variables.

**Specification**	**A-1**	**B-2**	**C-3**	**D-4**	**E-5**	**F-6**	**G-7**	**H-8**	**I-9**	**J-10**
**A-1**	1,00	0,32	-0,11	-0,13	0,49	0,23	0,66	-0,09	0,06	0,16
**B-2**	0,32	1,00	0,08	0,49	-0,04	0,06	0,29	0,54	0,34	0,68
**C-3**	-0,11	0,08	1,00	-0,10	0,03	-0,02	-0,08	-0,11	0,27	0,36
**D-4**	-0,13	0,49	-0,10	1,00	-0,25	-0,14	-0,20	0,98	-0,14	0,31
**E-5**	0,49	-0,04	0,03	-0,25	1,00	0,62	0,55	-0,27	-0,16	0,00
**F-6**	0,23	0,06	-0,02	-0,14	0,62	1,00	0,42	-0,15	-0,07	0,05
**G-7**	0,66	0,29	-0,08	-0,20	0,55	0,42	1,00	-0,12	0,10	0,22
**H-8**	-0,09	0,54	-0,11	0,98	-0,27	-0,15	-0,12	1,00	-0,04	0,40
**I-9**	0,06	0,34	0,27	-0,14	-0,16	-0,07	0,10	-0,04	1,00	0,48
**J-10**	0,16	0,68	0,36	0,31	0,00	0,05	0,22	0,40	0,48	1,00

Source: own work

Cluster analysis is also sensitive to the presence of outliers. This can result in a false group structure. To detect outliers, a case profile plot was utilized. In such a plot, outliers have distinctive profiles, often identified by extreme values of one or several variables. On the plot, some deviations were observed for certain cases, such as the Netherlands, but it only pertained to specific variables. It was decided not to exclude these cases from the analysis, as each case represented a single country, and removing it would eliminate the possibility of comparative analysis for European countries.

The data underwent standardization for cluster analysis. Standardization was not a necessary procedure since all variables were expressed in percentages. However, the decision was made to standardize the variables due to differences in their ranges. Standardization helps mitigate the impact of these differences, allowing for a more even treatment of each variable during analysis. Additionally, standardization was performed to address the influence of outliers. It can help alleviate the impact of outliers because the standard deviation considers outliers in a more moderate way than the range.

## Results and disscusion

### Financial problems with apartment payments

Financial situations impact the consumption behaviors of household members [34], including housing conditions. High financial burdens arising from housing costs can have adverse consequences for households [35]. The most immediate consequence is financial stress [36]. High housing costs limit households’ disposable income [37]. When a significant portion of a household’s income is allocated to housing costs, it can lead to continuous concerns about covering other essential expenses, such as food or healthcare [38]. This can result in reduced spending on higher-order needs or make it difficult for households to save for emergencies or retirement. Financial stress related to housing costs can have negative health effects, including increased anxiety levels and depression [39]. To cover housing costs, some households may resort to debt, such as credit card debt or personal loans. Accumulating debt can lead to a cycle of financial instability. Households burdened with high housing costs may be at risk of housing insecurity. They may struggle to pay rent or mortgage payments, which can lead to serious consequences such as eviction, exclusion, or homelessness.

High housing costs can contribute to the impoverishment of households, and even poverty [40]. In such conditions, investing in vocational training or starting a business becomes difficult. High housing costs can exacerbate wealth inequalities [41] because those who can afford housing investments benefit from the rising property values, while others struggle with rent or mortgage payments. Financial burdens caused by housing costs can also lead to social isolation.

According to Eurostat data, in 2022, the highest percentage of one-person households facing financial difficulties in covering housing costs was reported in Cyprus, 48%. In Croatia, Bulgaria, Spain, Greece, and Italy, the percentage of one-person households reporting these issues ranged from 40–43%. The lowest shares of one-person households with financial problems related to housing costs were in Estonia (7.3%), Latvia (10.9%), Sweden (14.3%), and Finland (15.1%). Significant changes occurred in the percentage of households with a heavy financial burden due to housing costs between 2005 and 2022. There was a statistically significant decrease in the percentage of one-person households facing significant financial difficulties in covering housing costs in Bulgaria, Germany, Estonia, Spain, France, Italy, Lithuania, Hungary, Malta, Austria, Poland, Slovenia, Finland, and Sweden. On the other hand, Denmark and Greece experienced a statistically significant increase in the percentage of one-person households with a heavy financial burden due to housing costs (Table 3).

**Table 3:** Linear trend analysis results for the share of one-person households with heavy financial burden due to housing costs in 2005–2022.

Specification	Slope coefficient of the trend (B)	p-value	R^2
Belgium	-0.233	0.3655	---
Bulgaria	-1.146	0.0016	0.4394
Czechia	-0.052	0.8465	---
Denmark	0.354	0.0021	0.4204
Germany	-1.052	0.0000	0.7725
Estonia	-0.455	0.0002	0.5766
Ireland	0.031	0.9125	---
Greece	1.110	0.0061	0.3452
Spain	-0.566	0.0456	0.1785
France	-0.436	0.0037	0.3812
Croatia	-0.562	0.1832	0.0521
Italy	-0.821	0.0209	0.2464
Cyprus	-0.588	0.1262	0.0862
Latvia	-0.466	0.2366	0.0292
Lithuania	-1.036	0.0225	0.2399
Luxembourg	0.144	0.5676	---
Hungary	-0.702	0.0423	0.1854
Malta	-2.057	0.0001	0.6164
Netherlands	-0.2254	0.2683	0.0182
Austria	-0.617	0.0000	0.7333
Poland	-0.875	0.0000	0.5433
Portugal	0.033	0.9241	---
Romania	-0.627	0.2484	0.0249
Slovenia	-1.278	0.0029	0.4003
Slovakia	0.399	0.1803	0.0535
Finland	-0.024	0.0116	0.2949
Sweden	-0.215	0.0064	0.3413

Source: own calculations based on Eurostat data.

Turning to the situation related to high financial burdens for housing in the V4 countries, it can be noted that the most unfavorable situation was in Slovakia. On the one hand, Slovak one-person households showed the highest percentage of individuals facing significant financial problems with housing costs. On the other hand, there was not statistically significant increase in the percentage of one-person households reporting these problems in recent years. In the Czech Republic, around 27% of one-person households reported problems with high housing expenses, although there was no statistically significant increase in the proportion of such households between 2005 and 2022. In Poland, despite a relatively high percentage of one-person households reporting housing cost problems (33%), there was a statistically significant decrease in the proportion of households reporting these issues. Similarly, in Hungary, there was a statistically significant decrease in one-person households burdened with high housing expenses. The percentage of individuals leading one-person households reporting significant financial problems was slightly over 24%, placing Hungary in the most favorable position among V4 Group countries. It is also worth mentioning that one of the reasons for financial burden may be the ratio of housing costs to gross income. In three V4 countries, the share of housing maintenance costs exceeded 20% of income. In Slovakia, it amounted to 27.4%, in the Czech Republic 23.4%, and in Poland (21.2%). Hungary, on the other hand, achieved a ratio below 20% (19.9%).

The housing situation in Slovakia has been highlighted by other researchers. In the study by Výbošťok & Štefkovičová [42], it was indicated that, on the one hand, after 2008, housing in Slovakia became more accessible due to rising incomes. On the other hand, the housing affordability index in Slovakia declined due to rising property prices.

To address the consequences of high household financial burdens related to housing costs, authorities often work on creating affordable housing options [43, 44], implementing rent control measures, providing financial assistance to low-income households [45], and promoting financial literacy to help households manage housing costs better [46, 47]. Initiatives for affordable housing construction, as well as the availability of mortgage loans and interest rate levels, aim to reduce households’ financial burdens and improve overall economic well-being [48].

### Thermal comfort issues

Problems with maintaining proper thermal conditions in residential spaces have serious consequences for households and are associated with a reduced quality of life. Inadequate thermal conditions, especially low indoor temperatures, can lead to various health issues such as colds, flu, or respiratory infections [49]. Older individuals, who often form one-person households, are particularly vulnerable to these risks. Difficulties in maintaining suitable thermal conditions can affect residents’ comfort. Low temperatures and a lack of thermal comfort can lead to both psychological and physical discomfort. In chilly or cold conditions, remote workers may struggle to concentrate and be productive, affecting their professional performance. Inadequate indoor temperatures can also lead to taking out loans or accumulating debt to finance the costs of maintaining proper thermal conditions at home, ultimately resulting in financial problems.

Maintaining favorable thermal conditions in residential spaces can be linked, among other things, to the technical aspects of buildings [50]. If a building is poorly insulated, households will need to spend more money on heating during the winter season. This can be a significant expense in household budgets, especially in regions with harsh climates. Inadequate thermal insulation can lead to condensation of water vapor and the growth of mold and moisture on walls, potentially causing damage to the building’s structure and requiring costly repairs. Leaky windows, doors, and inefficient thermal insulation contribute to heat loss, not only increasing energy bills but also having a negative impact on the environment. Poorly insulated buildings that struggle to maintain proper thermal conditions can lose value in the real estate market, affecting household investment capital. Increased nergy consumption for heating in poorly insulated buildings contributes to higher carbon dioxide emissions, further exacerbating climate change.

In 2022, the highest percentage of one-person households reporting an inability to maintain proper thermal conditions in their homes were Bulgarians, Greeks, and Lithuanians, with respective percentages of 31.8%, 26.8%, and 25.6%. At the same time, over 20% of one-person households faced issues with maintaining adequate indoor temperatures in Portugal and Spain. In contrast, Finns, Luxembourgers, and Swedes had the lowest percentages of households reporting problems with maintaining proper thermal conditions in residential spaces, with these households not exceeding 3.6%. From 2005 onwards, Poland, Bulgaria, and Portugal experienced the most significant improvement in thermal conditions in homes. Over the seventeen-year period, the percentage of households reporting difficulties in maintaining the appropriate indoor temperaturę in residential spaces decreased by 36.1 percentage points in Poland, 43.6 percentage points in Bulgaria, and 29.8 percentage points in Portugal, and these changes were statistically significant (p<0.05) (Table 4).

**Table 4:** Linear trend analysis results for the percentage of one-person households reporting problems with maintaining proper thermal conditions in residential spaces in 2005–2022.

Specification	Slope coefficient of the trend (B)	p-value	R^2
Belgium	-0.601	0.0005	0.5639
Bulgaria	-2.744	0.0000	0.9322
Czechia	-0.493	0.0000	0.7896
Denmark	-0.242	0.1512	0.0696
Germany	1.972	0.1378	0.0896
Estonia	0.034	0.6476	---
Ireland	0.030	0.7720	---
Greece	0.301	0.2026	0.0431
Spain	2.092	0.0972	0.1288
France	0.033	0.4076	---
Croatia	-0.356	0.0071	0.5237
Italy	2.205	0.0621	0.1717
Cyprus	-0.781	0.0003	0.5926
Latvia	0.800	0.5159	---
Lithuania	1.665	0.1002	0.1227
Luxembourg	2.302	0.0896	0.1342
Hungary	-0.602	0.0008	0.5359
Malta	-0.273	0.4164	---
Netherlands	0.047	0.1682	0.6900
Austria	-0.122	0.0003	0.5892
Poland	-2.011	0.0000	0.9186
Portugal	-1.734	0.0000	0.8183
Romania	-1.691	0.0000	0.8090
Slovenia	-0.040	0.5815	---
Slovakia	-0.200	0.1536	0.0784
Finland	-0.012	0.5511	---
Sweden	0.03	0.3168	0.0052

Source: own calculations based on Eurostat data.

Between 2005 and 2022, statistically significant improvements were observed in thermal conditions in residential spaces in other countries, in addition to the three mentioned above. A decrease in the percentage of one-person households reporting thermal issues was observed in countries such as Romania, Croatia, Belgium, Bulgaria, Hungary, Czech Republic, Cyprus, Portugal, Denmark, and Austria. In other countries, there were no statistically significant changes in the situation during the examined period. The highest rate of change was observed in Bulgaria, Poland, Portugal, and Romania. On average, year-on-year, the percentage of single-person households having problems with maintaining proper thermal conditions in residential spaces decreased from 1.7% in Romania to 2.7% in Bulgaria from 2005 to 2022.

In summary, regarding the Visegrad Group (V4) countries, the lowest percentage of one-person households reporting problems with maintaining proper thermal conditions was in the Czech Republic (5.2% in 2022). In other V4 countries, the percentages of one-person households with this issue were slightly higher, ranging from 7.9% in Hungary, to 8.7% in Poland. In all V4 countries, except Slovakia, there was a statistically significant decrease in the percentage of one-person households reporting problems with maintaining proper thermal conditions in residential spaces.

The issue of maintaining proper thermal conditions in residential spaces has an impact on the health, finances, and overall quality of life of households [51, 52]. Investments in energy efficiency and thermal insulation can help address these problems and improve housing conditions. Among the Visegrad Group (V4) countries, Slovakia has the worst position concerning the satisfaction of the thermal needs of household members, not only among single-person households [53]. Slovakia lags behind in progress in alleviating energy poverty. There are also doubts about the appropriateness of measures aimed at combating it. It is noted that the multidimensional nature and specificity of energy poverty in Slovakia are still inadequately addressed in key policies. Furthermore, there are shortcomings in policy aspects related to distribution and participation. Social welfare benefits are difficult to access for energy-poor households, as are energy efficiency support programs. While some policies consider dimensions related to this issue, it does not reflect the uneven occurrence of energy poverty [53].

In another scientific article [54] addressing energy efficiency in Poland and Slovakia, it is pointed out that low energy efficiency in single-family homes results in increased heating costs and, consequently, exacerbates the issue of energy poverty. This phenomenon involves homeowners not being able to afford energy or energy services that would allow them to maintain a suitable temperature while using good-quality fuel. Building energy efficiency translates to energy savings, and reducing energy production means not only financial savings but also reduced environmental pollution. However, it is noted that both in Slovakia and Poland, there is insufficient communication from the state (government policies) towards individuals and households to link increased energy efficiency with individual well-being.

Regarding the policies of both countries, it should be noted and emphasized that appropriate regulations and programs are being implemented to improve energy efficiency policies. In Poland and Slovakia, there is a significant gap in public knowledge about energy efficiency.

The EU has recently introduced a new ambitious policy to encourage member states to take action to improve the energy efficiency of buildings. New regulations take into account that the main obstacle to building renovation is costs, including those of buildings in the manufacturing sector. Currently, about 75% of buildings in the EU are energy-inefficient, meaning that a significant portion of our electricity consumption is wasted. Reducing energy losses is possible through the renovation of existing buildings and the use of intelligent solutions and energy-efficient materials in new construction. Improving the energy efficiency of buildings plays a crucial role in achieving the ambitious goal of carbon neutrality by 2050, as outlined in the European Green Deal strategy [55].

### Environmental pollution issues

Environmental pollution, dirt, or other environmental problems in a specific area can have various negative consequences for households. Exposure to pollutants such as air or water pollution can lead to a range of health problems for household members [56]. These can include respiratory issues, allergies, skin diseases, and even more serious conditions like cancer. Air pollution, such as smog or particulate matter, can lead to breathing problems, especially in older individuals who often form single-person households. This can result in increased healthcare costs. Environmental problems like noise, litter, and unpleasant odors can significantly reduce residents’ quality of life [57]. They can disrupt daily activities, sleep patterns, and overall well-being. Environmental issues can also negatively impact property values. Homes in areas with visible pollution or environmental degradation may be less attractive to potential buyers or renters, leading to a decline in property value. Households may incur higher cleaning and maintenance costs due to the need for more frequent cleaning and repairs caused by environmental problems. For example, pollution can lead to building soiling and increased wear and tear. Living in a polluted or environmentally problematic environment can cause mental stress. In particular, noise pollution can lead to increased stress levels, irritability, and sleep disturbances. Unsightly pollution and dirt can cause aesthetic discomfort. Residents may feel uncomfortable looking at environmental degradation or being in an environment affected by it, which can negatively affect their mood. Residents of areas with significant environmental problems may experience social isolation as they limit outdoor activities and interactions with neighbors to avoid pollution or discomfort. Costs associated with mitigating the effects of environmental problems, such as purchasing air purifiers or treating health problems, can lead to financial burdens for households.

The highest percentage of single-person households reporting problems with polluted or problematic environments was in Malta (30.8%) and Greece (21.7%). The lowest percentages of single-person households reporting issues with their surroundings were observed in Croatia (4.2%), Sweden (6.6%), and Ireland (6.8%). In most European countries between 2005 and 2020, statistically significant changes occurred in the percentage of single-person households reporting problems with environmental pollution in their place of residence, including Belgium, Bulgaria, and Italy, where the percentage decreased. The most significant statistically significant decrease in the percentage of single-person households reporting environmental issues in their place of residence between 2005 and 2020 occurred in Germany (11 pp), Cyprus (9.9 pp), the Czech Republic (8.7 pp), and Estonia (8.5 pp). The smallest statistically significant progress in this area was made in Ireland, with only a 0.7 pp improvement. In the Netherlands, however, a statistically significant increase in the percentage of people reporting problems with their environment in their place of residence was observed (Table 5).

**Table 5:** Linear trend analysis results for the share of single-person households reporting problems with pollution, grime or other environmental in 2005–2020.

Specification	Slope coefficient of the trend (B)	p-value	R^2
Belgium	-0.171	0.0357	0.2267
Bulgaria	-0.214	0.0043	0.4123
Czechia	-0.528	0.0001	0.6295
Denmark	0.107	0.2354	0.0346
Germany	-0.086	0.6607	---
Estonia	-0.734	0.0006	0.5521
Ireland	-0.187	0.0208	0.2780
Greece	-0.113	0.5919	---
Spain	-0.251	0.0373	0.2224
France	0.018	0.8924	---
Croatia			
Italy	-0.721	0.0000	0.8019
Cyprus	-0.930	0.0000	0.8400
Latvia	-1.101	0.0000	0.6935
Lithuania	-0.022	0.8077	---
Luxembourg	-0.025	0.8665	---
Hungary	-0.229	0.0507	0.1920
Malta	-0.521	0.0189	0.2871
Netherlands	0.139	0.0239	0.2653
Austria	0.071	0.2615	0.0240
Poland	-0.089	0.2803	0.0171
Portugal	-0.657	0.0000	0.6866
Romania	-0.271	0.0449	0.2044
Slovenia	-0.448	0.0000	0.7559
Slovakia	-0.654	0.0000	0.7481
Finland	-0.472	0.0004	0.5707
Sweden	-0.043	0.6720	---

Source: own calculations based on Eurostat data.

Considering the situation in the Visegrad Group (V4) countries, between 2005 and 2020, significant statistically significant decreases in the percentage of single-person households reporting problems with environmental pollution were observed in the Czech Republic and Slovakia. In Poland and Hungary, stabilization was observed in this area. In 2020, the situation was most favorable concerning these aspects in the Czech Republic and Slovakia, with 9.1% and 11.1% of single-person households reporting problems with environmental pollution, respectively. In Hungary, the percentage of single-person households with these issues was 12.2%, while in Poland– 14.5%.

To address the consequences of pollution and other environmental issues, communities and local authorities often collaborate to implement environmental regulations, improve waste management, reduce pollution, and enhance the overall quality of the environment in a given area. Awareness campaigns and social initiatives can also encourage responsible environmental behaviors and reduce the impact of pollution and dirt on households.

### Safety issues in residential areas

Another important aspect of housing conditions is safety in a given area. Living in an environment where people are exposed to crime, violence, or vandalism carries health consequences in the form of stress, anxiety, and a decline in well-being. In the case of violent crimes, households may directly experience physical injuries or property damage, resulting in medical expenses and property repair costs. Exposure to crime and violence can have long-lasting psychological effects on individuals and families, leading to trauma, fear, and emotional distress. An increase in crime or vandalism in the neighborhood can negatively affect the value of homes. Potential buyers or renters may be discouraged from moving to unsafe areas, leading to a decline in property values [58]. The overall quality of life for residents may deteriorate in areas with high levels of crime and violence [59]. People may limit outdoor activities and social interactions due to safety concerns. Crime can undermine community cohesion and trust among neighbors. Residents may become more isolated and less inclined to engage in social activities or mutual support, which is particularly detrimental to single-person households.

In 2020, the highest percentages of single-person households reporting problems related to crime, violence, or vandalism in the area were observed in Greece and the Netherlands, with 21.1% and 19.0%, respectively, reporting such issues. The lowest percentages in this group of households were reported in Croatia (2.6%) and Lithuania (3.9%). Between 2005 and 2020, all surveyed countries saw a decrease in the percentage of single-person households reporting problems with crime, violence, or vandalism in the area. The most significant changes occurred in Ireland with a decrease of 16.1 pp and in Latvia with a decrease of 13.9 pp. Statistically significant decreases were also observed in Belgium, the Czech Republic, Denmark, Estonia, Lithuania, Hungary, Slovenia, and Finland (Table 6).

**Table 6:** Linear trend analysis results for the share of single-person households reporting a problem with crime, violence or vandalism in the area in 2005–2020.

Specification	Slope coefficient of the trend (B)	p-value	R^2
Belgium	-0.526	0.0002	0.6160
Bulgaria	-0.163	0.2798	0.0173
Czechia	-0.623	0.0002	0.6207
Denmark	-0.603	0.0002	0.6286
Germany	-0.170	0.8765	---
Estonia	-1.071	0.0000	0.9269
Ireland	-0.083	0.0003	0.5879
Greece	0.296	0.2134	0.0444
Spain	-0.362	0.0242	0.2643
France	-0.180	0.0295	0.2452
Croatia	-7.93	0.0001	0.6321
Italy	-0.284	0.0331	0.2341
Cyprus	0.062	0.6630	---
Latvia	-1.445	0.0000	0.8273
Lithuania	-0.289	0.0001	0.6379
Luxembourg	0.060	0.5821	---
Hungary	-0.475	0.0035	0.4292
Malta	-0.036	0.6302	---
Netherlands	-0.049	0.6096	---
Austria	-0.376	0.0033	0.4330
Poland	-0.089	0.2803	0.0178
Portugal	-0.286	0.0125	0.3245
Romania	-3.833	0.0106	0.3389
Slovenia	-0.346	0.0001	0.6296
Slovakia	-0.110	0.3182	0.0047
Finland	-0.859	0.0000	0.7680
Sweden	0.117	0.3109	0.0070

Source: own calculations based on Eurostat data.

In the Visegrad Group (V4) countries, the situation was as follows. Poland (5.0%) and Hungary (6.2%) had the lowest percentages of single-person households reporting problems with crime, violence, or vandalism in the area. In the Czech Republic and Slovakia, the percentages of single-person households reporting these issues were 7.1% and 7.4%, respectively. Between 2004 and 2020, the Czech Republic and Hungary experienced statistically significant decreases in the mentioned group of households reporting problems with a dangerous environment, while Poland and Slovakia observed stabilization in this regard.

To mitigate the consequences of crime, violence, or vandalism in residential areas, communities and local authorities often collaborate on implementing measures to prevent these negative behaviors, improve police work and safety, and support crime victims [60]. Neighborhood watch programs, increased street lighting, and community efforts are examples of initiatives aimed at reducing crime and its impact on households. There are also reports suggesting that environmental greening has positive effects in combating crime [61].

### Ranking of European countries according to housing conditions in one-person households

**Fig 1:**
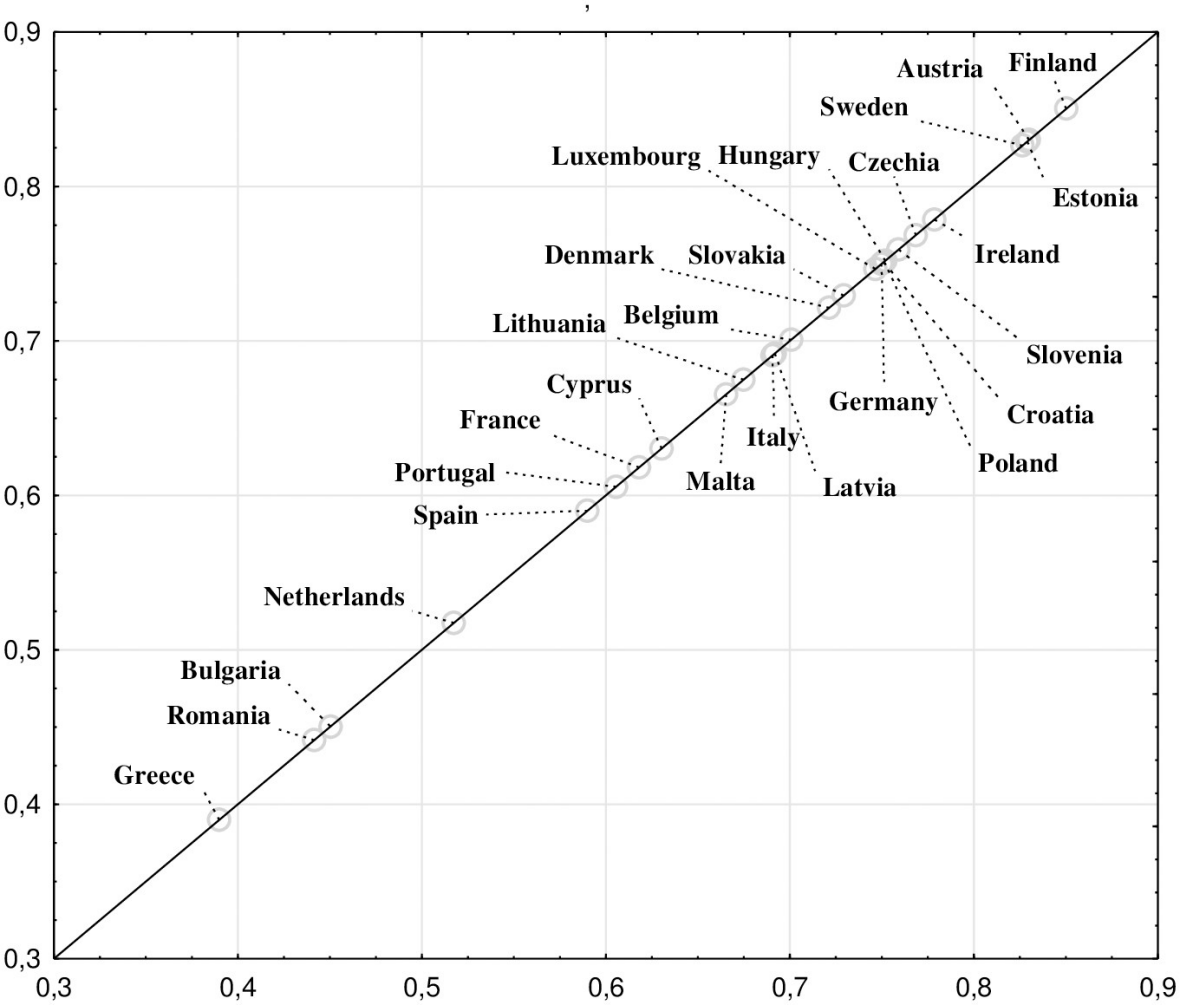
Ranking of European countries based on housing conditions in one-person households. Source: own work based on Eurostat database.

In the next step, the order of countries was determined based on 10 criteria (variables) indicated in the Data and Research Method section. The highest percentages of single-person households with poor housing conditions were observed in Greece, Romania, Bulgaria, and the Netherlands. Conversely, the lowest percentages of single-person households with inadequate housing conditions were found in Finland, Austria, Sweden, and Estonia. Among the Visegrád Group (V4) countries, Czech one-person households achieved the most favorable ranking, securing the 6th position among European countries. Slightly lower rankings were obtained by Hungary and Poland, ranking 8th and 9th, respectively. The highest housing deficiencies in single-person households were reported in Slovakia (13th position). So the order of the V4 countries in the obtained ranking corresponds to the order in the previously mentioned OECD Better Life Index ranking [28]. Generally, the Visegrád Group (V4) countries, in comparison to other European countries (a total of 27 countries were analyzed), exhibited relatively low percentages of one-person households reporting various housing-related problems (Fig 1).

Based on the Pearson correlation coefficient (r-Pearson) values between the percentage of one-person households in a country’s population and the rank value calculated for each country in the linear ordering analysis, it can be concluded that there is a weak correlation (0.247) between these two characteristics.

### Groups of countries by housing conditions in one-person households

**Fig 2:**
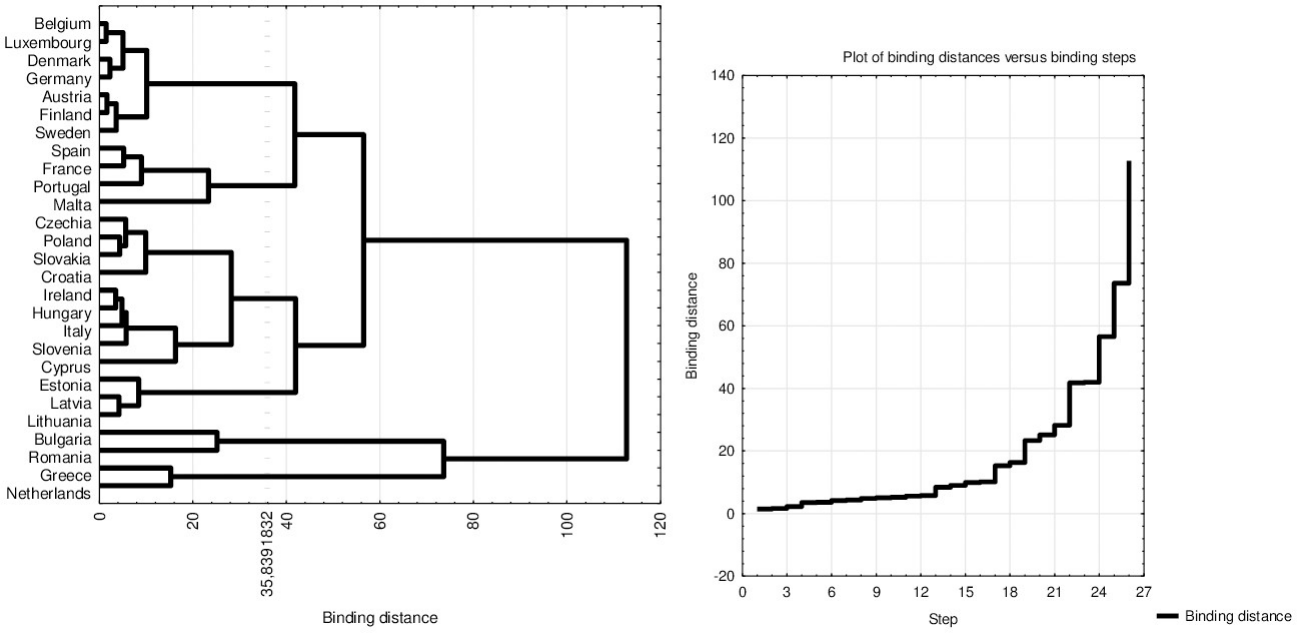
Groups of European countries based on housing conditions in single-person households. Source: own work based on Eurostat database.

As a result of grouping countries based on the percentage of single-person households indicating specific housing deficiencies, six groups were identified (Fig 2). In 1st group, we have Greece and the Netherlands. These countries had the highest housing cost overburden rate (61–71%) among European countries and the highest percentage of single-person households reporting problems with crime, violence, or vandalism in the area (19–21%).

In 2nd group, Bulgaria and Romania were noted. High percentages in these countries pertained to persons who cannot afford to replace worn-out furniture (54–57%) and the population having neither a bath nor a shower in their dwelling. In Romania, close to every third single-person household was not equipped with neither a bath nor a shower, and this was the highest value among all the analyzed countries. The first two clusters encompassed countries with the most unfavorable housing conditions in single-person households.

The 3rd group consisted of Baltic countries: Lithuania, Latvia, and Estonia. They were characterized by relatively low percentages of single-person households with a housing cost overburden rate (9–14%), a low percentage of households with a heavy financial burden due to housing costs (7–18%), and a low percentage of households reporting problems with noise from neighbors or from the street (7–14%). Conversely, relatively high percentages were observed regarding the absence of both a bath and a shower in their dwelling, ranging from 11% to 14% of all single-person households.

4th group included the Visegrád Group (V4) countries, as well as Cyprus, Slovenia, Italy, Ireland, and Croatia. In these countries, there were relatively high percentages of one-person households facing a significant financial burden due to housing costs (ranging from 20% in Slovenia to 48% in Slovakia and Cyprus–the highest percentage among all the analyzed countries). Additionally, relatively low percentages indicated issues with noise from neighbors or from the street in single-person households, ranging from 8% in Croatia to 17% in Cyprus. Interestingly, all V4 countries were in the same cluster, indicating similarities in meeting the needs of single-person households inadequately.

5th group consisted of Malta, Portugal, France, and Spain. These countries had relatively poor housing conditions in single-person households but were more favorable than in the first two clusters. Relatively low percentages of households indicated the population having neither a bath nor a shower in their dwelling (from 0,1% in Malta to 2% in Portugal), while relatively high percentages of households reported inconveniences related to noise from neighbors or from the street (from 23% in Spain to 29% in Malta).

The last group, 6th group, comprised Sweden, Finland, Austria, Germany, Denmark, Luxembourg, and Belgium. These countries had the most favorable housing conditions in single-person households (Table 7).

**Table 7:** Clustering of countries by unmet housing needs in single-person households (in % of households with unmet needs).

Specification	Housing cost overburden rate	Persons who cannot afford to replace worn-out furniture	Population living in a dwelling with a leaking roof, damp walls, floors or foundation, or rot in window frames or floor	Population having neither a bath, nor a shower in their dwelling	Noise from neighbours or from the street	Pollution, grime or other environmental problems	Crime, violence or vandalism in the area	Households with heavy financial burden due to the housing costs	Inability to keep home adequately warm
**1^st^ cluster**									
**Greece**	60,7	57,2	15,0	0,3	24,0	21,7	21,1	41,1	26,8
**Netherlands**	70,9	33	15,8	0,1	35,9	17,5	19,0	18,4	11,2
**2^nd^ cluster**									
**Bulgaria**	37,8	53,7	12,0	10,1	9,3	9,6	18,2	42,5	31,8
**Romania**	21,2	57,2	12,9	30,0	19	12,2	10,8	27,3	15,9
**3^rd^ cluster**									
**Estonia**	13,9	26,7	11,5	10,7	6,6	8,5	5,2	7,3	5,8
**Latvia**	13,8	35,8	15,9	12,4	14,1	14,1	6,6	10,9	13,4
**Lithuania**	8,9	27,3	11,9	13,8	14	12,9	3,9	17,5	25,6
**4^th^ cluster**									
**Czechia**	22,6	44,7	6,1	0,5	14	9,1	7,1	26,9	5,2
**Slovakia**	15,2	33,7	6,3	1,1	11,5	11,1	7,4	48,2	9,1
**Italy**	18,0	21,9	19,3	1,1	14,1	14,6	8,5	40,3	13,1
**Poland**	23,1	20,1	7,7	4,4	15,1	14,5	5,0	32,9	8,7
**Croatia**	15,1	27,5	14,4	3,7	8,0	4,2	2,6	42,6	17,3
**Ireland**	10,6	24,2	18,5	0,5	9,1	6,8	11,4	25,8	10,7
**Hungary**	20,7	27,4	24	1,6	9,0	12,2	6,2	24,2	7,9
**Slovenia**	13,9	13,1	25,0	0,5	16,6	16,3	8,4	20,3	6,0
**Cyprus**	11,5	25,2	35,9	1,0	17,3	7,6	6,4	48,1	18,6
**5^th^ cluster**									
**Spain**	19,5	29,1	19	0,5	23,2	12,4	14,9	41,9	20,8
**France**	20,2	32,8	15,6	0,7	23,5	19,1	17,1	24,3	14
**Malta**	9,5	17,4	9,7	0,1	28,8	30,8	12,9	25,7	8,5
**Portugal**	10,8	41,0	26,4	2,0	25,0	13,6	8,2	24,1	24
**6^th^ cluster**									
**Belgium**	27,9	21,5	16,0	0,9	17,6	12,7	13,0	30,5	6,8
**Denmark**	40,4	17,2	13,1	2,9	24,1	12,2	7,7	18,8	9,0
**Germany**	23,8	19,5	11,3	0,0	25,0	14,4	10,6	15,7	7,3
**Luxembourg**	29,2	14,1	11,5	0,4	19,2	14,4	13,7	23,5	3,4
**Austria**	16,1	10,2	8,6	1,1	19,1	9,1	7,7	21,9	4,7
**Finland**	14,1	16,1	4,3	0,5	16,7	11,4	10,3	15,1	2,4
**Sweden**	25,0	8,6	6,1	0,2	19,8	6,6	14,8	14,3	3,6

Source: own work based on Eurostat database

The country groups presented based on housing conditions partly align with other groupings, such as those concerning household consumption structure [62, 63] or access to new information technologies [64], where distinct clusters consist of Western European, Eastern European, and Southern European countries. Thus, a kind of division of European countries has been maintained for many years in various aspects related to the standard of living in households.

## Conclusions

The aim of the study was to assess the satisfaction of housing-related needs in single-person households from European countries, with particular emphasis on the Visegrád Group (V4) countries. Based on the results of the analysis, it can be concluded that housing conditions in single-person households are improving in most European countries, including those in the Visegrád Group. In the ranking of countries based on housing conditions in single-person households, the best positions among V4 countries are held by single-person households in the Czech Republic and Hungary. Slovakia occupies the lowest position, although all V4 countries are above the average housing conditions in the EU-countries.

Six groups of countries were identified based on housing conditions in single-person households. V4 countries were placed in one cluster, indicating similarities in unmet housing needs in one-person households. The most favorable housing conditions in Europe for single-person households were found in Finland, Austria, Sweden, and Estonia. Housing policies in these countries pertaining to one-person households should serve as an example for other European countries. In these countries, except for Austria, there is a high percentage of single-person households among the total households, which may have led to the development of better solutions for the housing conditions and protection of members of these households. The worst housing conditions, reflecting unmet housing needs in single-person households, were observed in Romania, Bulgaria, Greece, and the Netherlands.

Based on the conducted research, it is evident that there is a need for actions to address housing-related issues in single-person households. These actions may include investments in building energy efficiency, initiatives for affordable housing construction, and environmental policies. This is crucial for both the health and well-being of residents and for improving the overall state of the environment and the quality of community life.

## References

[1] Lytvyn, V., Vysotska, V., Mykhailyshyn, V., Peleshchak, I., Peleshchak, R., & Kohut, I. Intelligent System of a Smart House. 2019 3rd International Conference on Advanced Information and Communications Technologies (AICT), IEEE, July 2019, pp. 282–287.

[2] Kayser G. L., Rao N., Jose R., & Raj A. Water, sanitation and hygiene: measuring gender equality and empowerment. Bulletin of the World Health Organization, vol. 97, no. 6, 2019, p. 438. doi: 10.2471/BLT.18.223305 31210683 PMC6560376

[3] Fogel B. S. Psychological aspects of staying at home. In Aging in place, Routledge, 2019, pp. 19–28.

[4] Tinson A., & Clair A. Better housing is crucial for our health and the COVID-19 recovery. The Health Foundation, vol. 20, no. 11, 2020, pp. 1–25.

[5] Werdhiastutie A., Suhariadi F., & Partiwi S. G. Achievement motivation as antecedents of quality improvement of organizational human resources. Budapest International Research and Critics Institute-Journal (BIRCI-Journal) Volume, vol. 3, 2020, pp. 747–752. doi: 10.33258/birci.v3i2.886

[6] O’Shaughnessy B., Manning R. M., Greenwood R. M., Vargas-Moniz M., Loubière S., Spinnewijn F., et al. Home as a base for a well-lived life: Comparing the capabilities of homeless service users in housing first and the staircase of transition in Europe. Housing, Theory and Society, vol. 38, no. 3, 2021, pp. 343–364. doi: 10.1080/14036096.2020.1762725

[7] Piekut M. Jednoosobowe gospodarstwa domowe w Polsce: uwarunkowania funkcjonowania, tendencje zmian. CeDeWu, 2019. (in Polish)

[8] Piekut M. Jednoosobowe gospodarstwa domowe w teorii gospodarstwa domowego. Problemy Zarządzania-Management Issues, vol. 18, no. 1 (87), 2020, pp. 109–133. (in Polish)

[9] Distribution of households by household type and income lev-l—EU-SILC survey [ILC_LVPH04__custom_7551880]. Eurostat database, 23/09/2023, [https://ec.europa.eu/eurostat/databrowser/view/ILC_LVPH04__custom_7551880/default/table?lang=e.

[10] Vansteenkiste M., Ryan R. M., & Soenens B. Basic psychological need theory: Advancements, critical themes, and future directions. Motivation and emotion, vol. 44, 2020, pp. 1–31. doi: 10.1007/s11031-019-09818-1].

[11] Widagdo B., & Roz K. Hedonic shopping motivation and impulse buying: the effect of website quality on customer satisfaction. The Journal of Asian Finance, Economics, and Business, vol. 8, no. 1, 2021, pp. 395–405.

[12] Rojas M., Méndez A., & Watkins-Fassler K. The hierarchy of needs empirical examination of Maslow’s theory and lessons for development. World Development, vol. 165, 2023, p. 106185. doi: 10.1016/j.worlddev.2023.106185

[13] Hopper E. Maslow’s hierarchy of needs explained. ThoughtCo, 2020, pp. 1–3.

[14] Wang T. C., Chen W. T., Kang Y. N., Lin C. W., Cheng C. Y., Suk F. M., et al. Why do pre-clinical medical students learn ultrasound? Exploring learning motivation through ERG theory. BMC Medical Education, vol. 21, no. 1, 2021, pp. 1–9. doi: 10.1186/s12909-021-02869-4 34412610 PMC8375120

[15] McClelland D. Achievement motivation theory. In Organizational Behavior 1, Routledge, 2015, pp. 46–60.

[16] Reeve J., Ryan R., Deci E. L., & Jang H. Understanding and promoting autonomous self-regulation: A self-determination theory perspective. In Motivation and self-regulated learning, Routledge, 2012, pp. 223–244.

[17] Ryan R. M., & Deci E. L. Brick by brick: The origins, development, and future of self-determination theory. In Advances in motivation science, vol. 6, Elsevier, 2019, pp. 111–156. doi: 10.1016/bs.adms.2019.01.001

[18] Max-Neef M. Development and human needs. In Development ethics, Routledge, 2017, pp. 169–186. doi: 10.4324/9781315258003-14

[19] Keyes C. L. M., & Ryff C. D. Subjective change and mental health: A self-concept theory. Social psychology quarterly, 2000, pp. 264–279. doi: 10.2307/2695873

[20] Maslow A. H. A Dynamic Theory of Human Motivation. Psychological Review, vol. 50, 1958, pp. 370–396. doi: 10.1037/h0054346

[21] Maslow A., & Lewis K. J. Mas’ow’s hierarchy of needs. Salenger Incorporated, vol. 14, no. 17, 1987, pp. 987–990.

[22] Jenkins D., & Brownlee K. What a home does. Law and Philosophy, vol. 41, no. 4, 2022, pp. 441–468. doi: 10.1007/s10982-021-09414-w

[23] Pandiangan S. M. T. Analysis of Factors Affecting Interest in Buying a House. Journal of Innovation Research and Knowledge, vol. 2, no. 6, 2022, pp. 2615–2620.

[24] Pohl L., Genz C., Helbrecht I., & Dobrusskin J. Need for shelter, demand for housing, desire for home: A psychoanalytic reading of home-making in Vancouver. Housing Studies, vol. 37, no. 9, 2022, pp. 1650–1668. doi: 10.1080/02673037.2020.1857708

[25] Tanjitpiyanond P., Jetten J., & Peters K. How economic inequality shapes social class stereotyping. Journal of Experimental Social Psychology, vol. 98, 2022, p. 104248. doi: 10.1016/j.jesp.2021.104248

[26] Cho M. E., & Kim M. J. Smart homes supporting the wellness of one or two-person households. Sensors, vol. 22, no. 20, 2022, p. 7816. doi: 10.3390/s22207816 36298165 PMC9611916

[27] Bhattacharya S., & Mittal P. The impact of individual needs on employee performance while teleworking. Australasian Accounting, Business and Finance Journal, vol. 14, no. 5, 2020, pp. 65–85. doi: 10.14453/aabfj.v14i5.5

[28] Better Life Index. OECD, Access 2/1/2024, https://www.oecdbetterlifeindex.org/topics/housing/.

[29] Green Living Index 2022. Wunderflats, Access 10/1/2024, https://assets.website-files.com/60bdda50ac6f13216e571789/61e9681ca9ac421d67e8ce6c_Green%20Living%20Index%202022%20EN.pdf.

[30] Gender Statistics Database. European Institute for Gender Equality. Access 20/1/2024, https://eige.europa.eu/gender-statistics/dgs/indicator/ta_livcond_housing_genhousat__eqls_satisareas__accomm.

[31] Grabiński T. Koncepcja badań efektywności procedur porządkowania liniowego. Zeszyty Naukowe/Akademia Ekonomiczna w Krakowie, no. 181, 1984, pp. 5–35.

[32] Backhaus K., Erichson B., Gensler S., Weiber R., & Weiber T. Cluster analysis. In Multivariate Analysis: An Application-Oriented Introduction, Wiesbaden: Springer Fachmedien Wiesbaden, 2023, pp. 453–532. doi: 10.1007/978-3-658-40411-6_8

[33] Anderberg M. R. Cluster analysis for applications: probability and mathematical statistics: a series of monographs and textbooks (Vol. 19). Academic press, 2014.

[34] Lorentz A., Ciarli T., Savona M., & Valente M. The effect of demand-driven structural transformations on growth and technological change. Journal of Evolutionary Economics, vol. 26, 2016, pp. 219–246. doi: 10.1007/s00191-015-0409-5

[35] Kaygusuz K. Energy services and energy poverty for sustainable rural development. Renewable And Sustainable Energy Reviews, vol. 15, no. 2, 2011, pp. 936–947. doi: 10.1016/j.rser.2010.11.003

[36] Friedline T., Chen Z., & Morrow S. P. Families’ financial stress & well-being: The importance of the economy and economic environments. Journal of Family and Economic Issues, vol. 42, 2021, pp. 34–51. doi: 10.1007/s10834-020-09694-9 32837140 PMC7362317

[37] Piekut M., & Piekut K. Changes in Patterns of Consumer Spending in European Households. Sustainability, vol. 14, no. 19, 2022, p. 12794. doi: 10.3390/su141912794

[38] Deidda M. Economic hardship, housing cost burden and tenure status: Evidence from EU-SILC. Journal Of Family And Economic Issues, vol. 36, 2015, pp. 531–556. doi: 10.1007/s10834-014-9431-2

[39] Ahmad K., Erqou S., Shah N., Nazir U., Morrison A. R., Choudhary G., et al. Association of poor housing conditions with COVID-19 incidence and mortality across US counties. PloS one, vol. 15, no. 11, 2020, e0241327. doi: 10.1371/journal.pone.0241327 33137155 PMC7605696

[40] Piekut M. Patterns of energy consumption in Polish one-person households. Energies, vol. 13, no. 21, 2020, p. 5699. doi: 10.3390/en13215699

[41] Zhu Y., Yuan Y., Gu J., & Fu Q. Neoliberalization and inequality: disparities in access to affordable housing in urban Canada 1981–2016. Housing Studies, 2021, pp. 1–28. doi: 10.1080/02673037.2021.2004093

[42] Výbošťok J., & Štefkovičová P. Housing affordability, quality of life, and residential satisfaction in the Austrian cross-border suburban region of Bratislava, Slovakia. Moravian Geographical Reports, vol. 31, no. 1, 2023, pp. 2–13. doi: 10.2478/mgr-2023-0001

[43] Koetter T., Sikder S. K., & Weiss D. The cooperative urban land development model in Germany-An effective instrument to support affordable housing. Land Use Policy, vol. 107, 2021, p. 105481. doi: 10.1016/j.landusepol.2021.105481

[44] Czischke D., & van Bortel G. An exploration of concepts and policies on ’affordable housing’ in England, Italy, Poland and The Netherlands. Journal of Housing and the Built Environment, vol. 38, no. 1, 2023, pp. 283–303. doi: 10.1007/s10901-018-9598-1

[45] Anacker K. B. Introduction: Housing affordability and affordable housing. International Journal of Housing Policy, vol. 19, no. 1, 2019, pp. 1–16. doi: 10.1080/19491247.2018.1560544

[46] Rahman M., Isa C. R., Masud M. M., Sarker M., & Chowdhury N. T. The role of financial behavior, financial literacy, and financial stress in explaining the financial well-being of B40 group in Malaysia. Future Business Journal, vol. 7, no. 1, 2021, pp. 1–18. doi: 10.1186/s43093-021-00099-0

[47] Despard M. R., Friedline T., & Martin-West S. Why do households lack emergency savings? The role of financial capability. Journal of Family and Economic Issues, vol. 41, 2020, pp. 542–557. doi: 10.1007/s10834-020-09679-8 32837139 PMC7236434

[48] Byrne M. Generation rent and the financialization of housing: A comparative exploration of the growth of the private rental sector in Ireland, the UK and Spain. Housing Studies, vol. 35, no. 4, 2020, pp. 743–765. doi: 10.1080/02673037.2019.1632813

[49] Palacios J., Eichholtz P., Kok N., & Aydin E. The impact of housing conditions on health outcomes. Real Estate Economics, vol. 49, no. 4, 2021, pp. 1172–1200. doi: 10.1111/1540-6229.12317

[50] Fernández-Agüera J., Domínguez-Amarillo S., Alonso C., & Martín-Consuegra F. Thermal comfort and indoor air quality in low-income housing in Spain: The influence of airtightness and occupant behavior. Energy and Buildings, vol. 199, 2019, pp. 102–114. doi: 10.1016/j.enbuild.2019.06.052

[51] Zhang Y., Wang H., Gao W., Wang F., Zhou N., Kammen D. M., et al. A survey of the status and challenges of green building development in various countries. Sustainability, vol. 11, no. 19, 2019, p. 5385. doi: 10.3390/su11195385

[52] Zhu W., Zhang Z., Li X., Feng W., & Li J. Assessing the effects of technological progress on energy efficiency in the construction industry: a case of China. Journal of Cleaner Production, vol. 238, 2019, p. 117908. doi: 10.1016/j.jclepro.2019.117908

[53] Koďousková H., & Bořuta D. Energy poverty in Slovakia: Officially defined but misrepresented in major policies. Energy Policy, vol. 168, 2022, p. 113095. doi: 10.1016/j.enpol.2022.113095

[54] Barwińska Małajowicz A., Knapková M., Szczotka K., Martinkovičová M., & Pyrek R. Energy Efficiency Policies in Poland and Slovakia in the Context of Individual Well-Being. Energies, vol. 16, no. 1, 2022, p. 116. doi: 10.3390/en16010116

[55] Perissi I., & Jones A. Investigating European Union decarbonization strategies: Evaluating the pathway to carbon neutrality by 2050. Sustainability, vol. 14, no. 8, 2022, p. 4728. doi: 10.3390/su14084728

[56] Chin Y. S. J., De Pretto L., Thuppil V., & Ashfold M. J. Public awareness and support for environmental protection-A focus on air pollution in peninsular Malaysia. PloS one, vol. 14, no. 3, 2019, p. e0212206. doi: 10.1371/journal.pone.0212206 []. 30870439 PMC6417846

[57] Alonso A., Patricio J., Suarez R., & Escandon R. Acoustical retrofit of existing residential buildings: Requirements and recommendations for sound insulation between dwellings in Europe and other countries worldwide. Building and Environment, vol. 174, 2020, p. 106771. doi: 10.1016/j.buildenv.2020.106771

[58] Ceccato V., & Wilhelmsson M. Do crime hot spots affect housing prices? Nordic journal of criminology, vol. 21, no. 1, 2020, pp. 84–102. doi: 10.1080/2578983X.2019.1662595

[59] Streimikiene D. Quality of life and housing. International Journal of Information and Education Technology, vol. 5, no. 2, 2015, p. 140. doi: 10.7763/IJIET.2015.V5.491

[60] Braga A. A., Turchan B., Papachristos A. V., & Hureau D. M. Hot spots policing of small geographic areas effects on crime. Campbell systematic reviews, vol. 15, no. 3, 2019, e1046. doi: 10.1002/cl2.1046 []. 37133274 PMC8356500

[61] Pizarro J. M., Sadler R. C., Goldstick J., Turchan B., McGarrell E. F., & Zimmerman M. A. Community-driven disorder reduction: Crime prevention through a clean and green initiative in a legacy city. Urban Studies, vol. 57, no. 14, 2020, pp. 2956–2972. doi: 10.1177/0042098019892163

[62] Piekut M., & Piekut K. (2022). Changes in Patterns of Consumer Spending in European Households. *Sustainability*, 14(19), 12794. doi: 10.3390/su141912794

[63] Piekut M. (2013). Konsumpcja w polskich gospodarstwach domowych na tle krajów europejskich. *Problemy Zarządzania*, (1/2013 (40) t. 1), 23–39.

[64] Piekut M., & Rybaltowicz J. (2024). The Role of Information and Communication Technologies in Rural Development/Rola technologii informacyjno-komunikacyjnych w rozwoju obszarów wiejskich. *Zagadnienia Ekonomiki Rolnej*, (378 (1)). doi: 10.30858/zer/181136

